# Abatacept inhibits progression of structural damage in rheumatoid arthritis: results from the long-term extension of the AIM trial

**DOI:** 10.1136/ard.2007.085084

**Published:** 2007-11-17

**Authors:** H K Genant, C G Peterfy, R Westhovens, J-C Becker, R Aranda, G Vratsanos, J Teng, J M Kremer

**Affiliations:** 1 University of California, San Francisco, San Francisco, California, USA; 2 Synarc, Inc., San Francisco, California, USA; 3 UZ Gasthuisberg, Leuven, Belgium; 4 Global Clinical Research, Immunology, Bristol-Myers Squibb, Princeton, New Jersey, USA; 5 Albany Medical College and The Center for Rheumatology, Albany, New York, USA

## Abstract

**Objective:**

Assess the effect of abatacept on progression of structural damage over 2 years in patients with rheumatoid arthritis who had an inadequate response to methotrexate.

**Methods:**

539 patients entered an open-label extension of the AIM (Abatacept in Inadequate responders to Methotrexate) trial and received abatacept. Radiographic assessment of the hands and feet was performed at baseline, year 1 and year 2. At year 2, each patient’s radiographs were scored for progression blinded to sequence and treatment allocation.

**Results:**

In patients treated with abatacept for 2 years, greater reduction in progression of structural damage was observed in year 2 than in year 1. The mean change in total Genant-modified Sharp scores was reduced from 1.07 units in year 1 to 0.46 units in year 2. Similar reductions were observed in erosion and joint space narrowing scores. Following 2 years of treatment with abatacept, 50% of patients had no progression of structural damage as defined by a change in the total score of ⩽0 compared with baseline. 56% of patients treated with abatacept had no progression during the first year compared with 45% of patients treated with placebo. In their second year of treatment with abatacept, more patients had no progression than in the first year (66% vs 56%).

**Conclusions:**

Abatacept has a sustained effect that inhibits progression of structural damage. Furthermore, the mean change in radiographic progression in patients treated with abatacept for 2 years was significantly lower in year 2 versus year 1, suggesting that abatacept may have an increasing disease-modifying effect on structural damage over time.

Rheumatoid arthritis (RA) is a chronic autoimmune disease that usually leads to synovial joint damage and consequent disability. Activated T cells play a crucial part in the immunopathology of RA.^1^ T cell proliferation and interaction with other cell types, including synovial macrophages, fibroblasts and B cells, result in the production of proinflammatory cytokines and matrix metalloproteinases that promote synovitis and erosive loss of subchondral bone. Thus, targeting T cell activation is a rational therapeutic approach for the treatment of RA.

T cell activation requires antigen recognition by the T cell receptor, referred to as signal 1, as well as a co-stimulatory signal, referred to as signal 2.^2^  One of the best characterised co-stimulatory pathways involves the interaction of CD28 expressed on T cells with CD80 and CD86 expressed on antigen-presenting cells. Endogenous down-regulation of CD28-mediated T cell co-stimulation occurs through T cell expression of cytotoxic T lymphocyte antigen 4 (CTLA-4). CTLA-4 binds to CD80 and CD86 with higher avidity than CD28, thus preventing co-stimulation through this pathway, and is a major down-regulatory signal for T cells.

Abatacept is a soluble, recombinant, fully human fusion protein composed of the extracellular domain of human CTLA-4 and the Fc domain of human IgG1, which has been modified to prevent complement fixation. Abatacept is the first in a class of biological agents that target the second signal required for full T cell activation, a mechanism of action that is fundamentally different than any other current biological RA therapy. The efficacy of abatacept monotherapy has been shown in a phase IIa trial in patients with RA with an inadequate response to disease-modifying anti-rheumatic drugs (DMARDs).^3^ The efficacy of abatacept added to methotrexate (MTX) was demonstrated in the phase IIb and phase III trials of patients with an inadequate response to MTX or tumour necrosis factor-targeting agents.^4^^–^6

Results from the 1-year phase III, randomised, double-blind trial of Abatacept in Inadequate responders to Methotrexate (AIM) trial have been previously reported.^5^ Abatacept added to MTX therapy resulted in clinically significant improvements in the signs and symptoms of RA, physical function and health-related quality of life in patients with an inadequate response to MTX. Further, abatacept resulted in a statistically significant slowing of the progression of structural damage after 12 months of treatment.

Progressive structural damage is associated with increasing disability over time.^7^ ^8^ Thus, the effects of abatacept treatment on radiographic outcomes were assessed over longer-term treatment. Here we present radiographic assessments after 2 years of abatacept treatment in an open-label extension of the AIM trial. These data demonstrate that radiographic progression is significantly inhibited over the 2 years. The maintenance of efficacy of abatacept therapy on other clinical end points will be described in full in another report.

## PATIENTS AND METHODS

The study design, baseline characteristics and results of the 12-month double-blind phase of this trial have been reported previously.^5^ Institutional review boards or independent ethics committees approved the clinical protocol, and written informed consent to the study protocol was provided by each patient.

### Patients

Patients eligible to participate in the AIM trial were at least 18 years of age, met the American Rheumatism Association criteria for RA,^9^ and had RA for at least 12 months. All patients were screened for tuberculosis. At randomisation, all patients had persistent, active RA despite treatment with MTX, with ⩾10 swollen joints, ⩾12 tender joints and a C-reactive protein concentration of ⩾1.0 mg/dl. Patients were required to have received MTX therapy at a minimum dose of 15 mg/week for at least 3 months, with the dose having been stable for a minimum of 28 days prior to randomisation. A 28-day washout period was required for all other DMARDs. Reduction of MTX dosage in the first 6 months was permitted only in cases of toxicity. Stable dosages of nonsteroidal anti-inflammatory drugs or corticosteroids at ⩽10 mg prednisone/day were allowed, with a stable dosage for 25 days prior to randomisation. Adjustment of MTX and/or corticosteroid dosages were permitted after month 6, as was treatment with an additional DMARD if deemed appropriate by the investigator, including hydroxychloroquine, sulfasalazine, gold or azathioprine.

### Study protocol

During the first year, patients were randomised to receive a fixed dose of abatacept of approximately 10 mg/kg body weight or placebo in a 2:1 ratio. The dose of abatacept was 500 mg for patients weighing less than 60 kg, 750 mg for patients weighing 60–100 kg, and 1 g for patients weighing more than 100 kg. Study medication was infused intravenously over 30 min on days 1, 15 and 29, and every 28 days subsequently. Patients completing the double-blind period were eligible to enter an open-label, long-term extension and receive abatacept therapy every 28 days at a dose of approximately 10 mg/kg, as described above. The loading dose used in the double-blind period was not utilised here. No premedication was required for the intravenous infusions.

### Radiographic evaluation

Radiographic assessments of hands, wrists and feet were performed at baseline, year 1 and year 2, or upon early termination. The trial was powered for radiographic findings at year 1, and the primary end point was the change in erosion score, with secondary assessments of joint space narrowing (JSN) and total score using the Genant-modified Sharp scoring system.^5^ For each patient, baseline, year 1 and year 2 radiographs were all re-read at year 2 by two independent expert readers blinded to the original treatment allocation and the sequence of films.^10^

**Figure 1:**
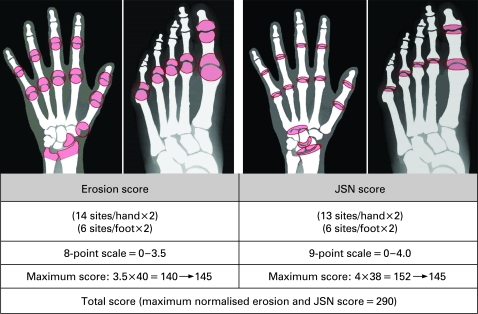
Genant-modified Sharp scoring system. Coloured areas indicate sites used to evaluate erosion and joint space narrowing (JSN) scores. Each site was evaluated in 0.5-unit increments as indicated. Erosion and JSN scores are normalised to 145 for a maximum total score of 290.

The Genant-modified Sharp scoring system (fig 1) assesses changes in structural damage, assigning scores for erosions of 0–3.5 (eight gradations) for 14 sites in each hand and wrist, and six sites in each foot.^11^ ^12^ JSN scores of 0–4 (nine gradations) are assigned to 13 sites in each hand and six sites in each foot.^12^ The erosion and JSN scores are normalised to 145 for a maximum total Genant-modified Sharp score of 290.^12^ Inter-reader, intra-class correlation coefficient (ICC) was determined based on the baseline and year 1 readings.^10^ Smallest detectable difference was determined as 1.96 SD of the two independent baseline readings.^13^

### Statistical analysis

The study was powered for change from baseline in the Genant-modified Sharp erosion score and used a rank-based analysis of covariance to compare changes between treatment groups at year 1. The radiographic analyses were performed on a modified intent-to-treat population at year 2. The modified intent-to-treat population included patients who were randomised and treated, completed the 1-year double-blind period, and continued to receive at least one dose of study medication during the open-label period. Patients who discontinued abatacept treatment during the open-label period were requested to return for follow-up radiography at year 2, regardless of any subsequent new anti-rheumatic therapy prescribed by the investigator. Radiographs were taken at the time of discontinuation in patients who were unwilling or unable to return at year 2. In these patients, 2-year data were imputed using linear extrapolation of the scores of the discontinuation film.

The changes from baseline in total Genant-modified Sharp scores, erosion scores and JSN scores were charted as cumulative probability plots to visually represent the distribution of all radiographic data. The observed cumulative proportion (scores ranked from the lowest to the highest and presented as a cumulative proportion of all scores) was plotted against the actual change from baseline of each score. The proportion of observations that fall below each possible change from baseline (*y*-axis) can be read on the *x*-axis.^14^

## RESULTS

### Patients and study completion

Of the 652 patients randomised and treated in the double-blind portion of the AIM trial, 433 received abatacept and 219 received placebo (table 1). As previously reported, baseline demographics and clinical characteristics did not differ significantly between patients in the abatacept and placebo groups.^5^ After the 12-month double-blind treatment period, all patients were eligible to receive open-label treatment with abatacept. More patients receiving abatacept treatment (89%) completed the 1-year double-blind phase relative to the placebo group (74%). Of the 547 patients who completed 12 months of treatment, 539 (83% of all randomised and treated patients) were treated with abatacept in the open-label period (378 initially randomised to abatacept (87%) and 161 to placebo (73.5%)). A high retention rate was maintained with open-label abatacept treatment, with 90% of the patients who entered the long-term extension completing 2 years.

**Table 1:** Number of subjects with radiographic assessments on days 365 and 729

	Abatacept n = 376 (%)	Placebo n = 160 (%)
Subjects included in analyses		
Day 365	328 (87.2)	144 (90.0)
Baseline and day 365	328 (87.2)	144 (90.0)
Day 729	324 (86.2)	143 (89.4)
Baseline and day 729	315 (83.8)	139 (86.9)
Imputation on day 729*	9 (2.4)	4 (2.5)
Subjects not included in the analyses		
No day 365 value†	2 (0.5)	1 (0.6)
No day 729 value‡	6 (1.6)	2 (1.3)

*Subjects are discontinued and qualified for imputation requirement. †Two subjects in the abatacept group had evaluative radiographic assessment outside the pre-specified visit window of day 365, and one subject in the placebo group had no radiographic assessment on day 365. ‡Subjects had evaluative radiographic assessment outside the pre-specified visit window of day 729.

Two-year radiographic data were available from 87% of patients who entered the open-label period, which comprises 72% of all randomised and treated patients (467 of 652 patients; table 1). Observed data were available for 97% of patients who completed the open-label period; 454 of 467 patients had radiographs at baseline and at year 2. Baseline and an early termination film were available for the remaining 3% of patients, and 2-year data were imputed by linear extrapolation. The data include baseline and year 2 radiographs of 324 patients treated with abatacept for 2 years (observed data in 315 patients, imputed data in nine patients). Therefore, the 2-year data represent the majority of randomised patients. As previously reported in an abstract,^10^ a high degree of inter-observer agreement was demonstrated by ICC for radiographic assessments at baseline (0.90) and 12 months (0.92) and for change between baseline and 12 months (0.82). The smallest detectable differences were 3.5, 2.5 and 5.1, respectively, for erosion, JSN and total score. These correspond to 1.9%, 1.3% and 1.3%, respectively, of the maximum values for each of these scores and compare favourably with values reported for other studies.^15^ ^16^

### Radiographic results: population-level data

**Figure 2:**
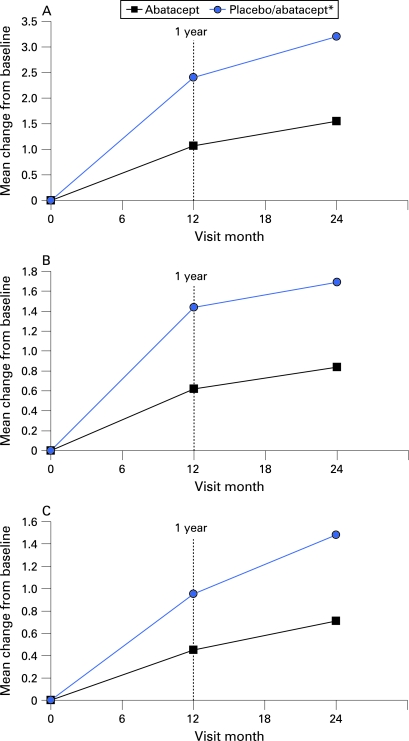
Mean change from baseline in total (A), erosion (B) and joint space narrowing (C) Genant-modified Sharp scores for patients at 12 months and 2 years. *Placebo patients were switched to abatacept after 12 months. All patients received background methotrexate.

The progression of structural damage was significantly reduced in patients treated with abatacept over 2 years versus those treated with placebo initially. Baseline erosion and JSN scores were comparable in patients originally randomised to either treatment group. Significantly lower mean changes in radiographic progression were observed in patients treated with abatacept for 2 years, with mean changes in total score, erosion and JSN scores of 1.55, 0.84 and 0.71 units, respectively, compared with 3.17, 1.48 and 1.69 units in patients treated with placebo for 12 months prior to abatacept open-label therapy (fig 2, table 2).

**Table 2:** Yearly mean changes in Genant-modified Sharp scores

	Δ Baseline to year 1	Δ Baseline to year 2	Δ Year 1 to year 2
Total score			
Abatacept	1.07	1.55	0.46
Placebo/abatacept	2.40	3.17	0.75
Erosion score			
Abatacept	0.62	0.84	0.21
Placebo/abatacept	1.44	1.69	0.25
Joint space narrowing score			
Abatacept	0.45	0.71	0.24
Placebo/abatacept	0.95	1.48	0.50

Baseline is day 1 of study. All randomised and treated patients who entered the open-label period and had radiographs at baseline and year 1. Baseline and year 1 radiographs were re-read at day 729. Placebo patients were switched to abatacept in the long-term extension (year 2). All patients received background methotrexate.

**Figure 3:**
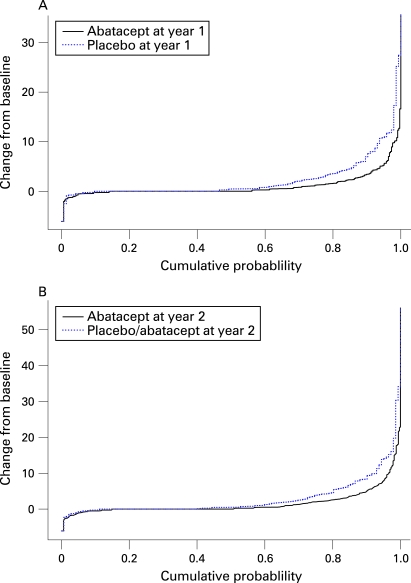
Cumulative probability distribution of changes from baseline in Genant-modified total Sharp scores by treatment at year 1 (A) and year 2 (B). The solid line represents patients treated with abatacept (A,B) and the dotted line represents patients treated with placebo (A) or treated with placebo for 12 months then abatacept for 12 months (B). All patients received background methotrexate.

Patients treated with abatacept for 2 years also had lower median changes in radiographic progression scores. The median change from baseline in total Genant-modified Sharp scores was 0.0 units at year 1 and year 2 with abatacept treatment (fig 3), versus median changes of 0.46 and 0.51 units at year 1 and year 2, respectively, in patients who received placebo prior to abatacept therapy. Further, the median change in erosion score was 0.0 units in patients treated with abatacept for 2 years, compared with 0.26 units in patients originally in the placebo group.

The mean change in the total score, erosion and JSN score were lower from year 1 to year 2 (0.46, 0.21 and 0.24 units, respectively) than the mean change in these scores from baseline to year 1 (1.07, 0.62 and 0.45 units, respectively) in patients treated with abatacept for up to 2 years (table 2). The increasing effect of treatment was consistently observed for both erosions and JSN.

### Radiographic results: patient-level data

Radiographic results at 2 years demonstrate the maintenance of effect of abatacept and MTX on non-progression of structural damage in patients with no progression at year 1 (table 3), defined as a change from baseline in total Sharp score of ⩽0. Overall, 50% of patients treated with abatacept did not progress from baseline to 2 years of treatment (163 of 324 patients). At 2 years, non-progression in erosions and JSN (change ⩽0) was observed from baseline in 56% and 68% of these patients, respectively. In patients treated with abatacept with no radiographic progression at year 1, 79% had no radiographic progression at year 2. Non-progression in erosions and JSN was maintained at 2 years in 83% and 87% of patients with no progression at 12 months.

**Table 3:** Patients with no progression in structural damage measured by Genant-modified Sharp scores in patients on abatacept therapy for 2 years

	Percentage of patients with no progression in structural damage*			Percentage of patients with no progression in structural damage in year 2†	
Outcome	Baseline to year 1 (n = 328)	Baseline to year 2 (n = 324)		Non-progressors at end of year 1 remaining non-progressors†	Progressors at end of year 1 becoming non-progressors
Erosion (%)	61	56		83	49
Joint space narrowing (%)	74	68		87	52
Total (%)	56	50		79	45

All patients received background methotrexate.

*Defined by a change in the total score of ⩽0 from baseline to year 1. †Defined by a change in the total score of ⩽0 from year 1 to year 2.

Non-progression at year 2 was also achieved in patients with initial damage progression on abatacept therapy. Of patients treated with abatacept who demonstrated radiographic progression at year 1, 45% had no progression of structural damage at year 2 (64 of 142 patients).  Further, 53% of patients originally randomised to placebo with progression at year 1 did not progress at year 2 after receiving abatacept treatment (42 of 79 patients).

Cumulative probability plots showing the distribution of change from baseline in total Genant-modified Sharp scores for year 1 and year 2 are shown in fig 3. Comparison of the curves in patients treated in the abatacept group (solid line) versus patients originally randomised to placebo (dotted line) demonstrates that abatacept treatment is associated with results in decreased numbers of patients with progression of structural damage. Furthermore, the amount of structural damage in patients who progressed is lower in patients treated with abatacept for both year 1 and year 2.

## DISCUSSION

Abatacept has been shown to significantly reduce disease activity in patients with RA. The previously described randomised, double-blind, placebo-controlled portion of the phase III study of abatacept in patients with RA with an inadequate response to MTX demonstrated statistically significant improvement in the signs and symptoms of disease, physical function assessed by the health-assessment questionnaire disability index, and health-related quality of life.^5^ In addition, abatacept positively impacted the rate of structural damage progression at 12 months, reducing it by approximately 50% compared with placebo. This placebo-controlled radiographic finding from randomised clinical trials provides an assessment of short-term effects on structural damage. However, only long-term radiographic progression has been associated with physical disability.^8^ Therefore, it is important to assess longer-term effects on structural damage beyond 1 year of therapy.

The retention rate of patients receiving abatacept treatment was high, with 89% completing the 1-year double-blind portion of the AIM trial,^5^ and 90% of the patients entering the long-term extension completing year 2, suggesting the tolerability and durability of response to abatacept. Further, radiographic data were collected from a high percentage of patients, with 2-year data available from 87% of patients entering the open-label period, and observed data for 97% of patients; linear imputation based on baseline and early termination films was used in only 3% of patients.

Of note, an increased proportion of patients randomised to the placebo group withdrew from the double-blind portion of the study (between baseline and year 1) due to lack of efficacy (18% vs 3% in the abatacept group).^5^ Patients initially randomised to placebo who discontinued the study during the double-blind period are not included in these calculations. As these patients with worsening disease were not included in the assessment of radiographic outcomes, the progression of structural damage in patients initially randomised to placebo may be underestimated. As is standard for the assessment of radiographic outcome, readers were blinded to the sequence of radiographs, thus eliminating bias for the expectation of benefit.

Abatacept therapy resulted in inhibition of structural damage progression over time. After 2 years of treatment, a significant reduction in the progression of structural damage was observed in patients treated with abatacept for 2 years relative to placebo for 12 months plus abatacept for 12 months.^8^ Lower mean changes in total Genant-modified Sharp scores at both 12 months and year 2 were 1.07 vs 2.4 units, and 1.55 vs 3.17 units, respectively. Notably, abatacept had a clear benefit on both the erosion and JSN scores.

The treatment effect for abatacept relative to placebo was an approximately 50% reduction in mean progression of structural damage in the first year of the study. The rate of progression of structural damage when patients had received placebo treatment for 2 years can be estimated by linear extrapolation from the 12-month data of patients randomised to placebo. Patients in the placebo group progressed >2 units in year 1 and can be projected to progress 4–5 units over 2 years using linear extrapolation. Overall, patients receiving abatacept treatment for 2 years progressed approximately 1.5 units versus the expected progression of approximately 4.5 units if placebo treatment had been continued for the second year. Thus, the effect of abatacept treatment in the second year of treatment would hypothetically reduce radiographic progression by an estimated two-thirds when compared with patients with established disease receiving MTX alone.

Fewer abatacept-treated patients had progression of structural damage, and, overall, 50% of patients receiving abatacept did not have radiographic progression over 2 years. In addition, 79% of patients treated with abatacept with no progression at year 1 maintained no progression of structural damage at year 2, demonstrating a durable effect on radiographic outcomes with abatacept therapy. In addition, 45% of patients with some progression at year 1 had no progression in year 2. As the overwhelming number of patients entering the second year of treatment completed the year, we are not reporting on a selected group of responders.

The increasing effect of abatacept treatment on the progression of structural damage is consistent with other efficacy data; the proportion of patients with American College of Rheumatology (ACR) 50 and 70 responses were statistically significantly increased from 6 months to 12 months in this study.^5^ The ACR 20, 50 and 70 responses; improvement in physical function; and improvement in both the physical and mental components of the Short-Form-36 health-related quality of life scale in patients treated with abatacept for up to 3 years will be described in full in a future report.

It is difficult to compare results across clinical trials with other treatments that have described radiographic outcomes. Confounding factors include the treatment and disease history of each study population, differing rates of radiographic progression for the control group in each trial, and the use of different scoring systems for the measurement of radiographic damage. The current study assessed the efficacy of abatacept in patients with established RA and long duration of disease, whereas several trials of other biological therapies, such as those targeting tumour necrosis factor, involved patients with early RA (eg, the ERA trial of etanercept^17^ and the PREMIER trial of adalimumab^18^) or MTX-naive patients (the TEMPO trial of etanercept^19^). The Sharp-modified Sharp and the van der Heijde-modified Sharp scoring systems utilised in the trials of tumour necrosis factor-targeting agents,^16^ ^17^ ^20^ as well as the Genant-modified Sharp scoring system utilised here and in the interleukin-1 receptor antagonist and rituximab studies,^18^ ^21^ all have differences in joints assessed, scales applied and maximum achievable scores. Thus, Sharp-, van der Heijde- and Genant-modified Sharp scores cannot be directly inter-converted, although they correlate moderately well cross-sectionally and longitudinally. In addition, the definition of the progression of structural damage varied among studies, including progression defined as change ⩾0 or the smallest detectable difference among readers.^16^^–^18 20 21 The Genant-modified scoring system employed in this study showed high inter-reader agreement (0.90–0.92 cross-sectionally and 0.82 for change) and high sensitivity to change (smallest detectable difference) for erosion (3.5), JSN (2.5) and total score (5.1).^10^

The inhibition of progression of structural damage with abatacept has clinical implications for the prevention of disability over time. The association of progression of structural damage and impaired physical function over the long term has been well correlated, and is strongest in established disease.^7^ ^8^ Impacting the rate of joint destruction with continued abatacept treatment is likely to impede progressive functional disability, and thus may have increasing benefit for patients over time. Further study in this and other patient populations will assess the efficacy of abatacept therapy over longer periods of time.
